# Gain and Loss of Heterozygosity in the Genome of the Asexual Nematode *Halicephalobus mephisto*

**DOI:** 10.1007/s00239-025-10259-3

**Published:** 2025-08-06

**Authors:** Ali Amini, John R. Bracht

**Affiliations:** 1 Center for Data Science, American University, 4400 Massachusetts Avenue, NW, Washington, DC 20016 USA; 2 Biology Department, American University, 4400 Massachusetts Avenue, NW, Washington, DC 20016 USA

**Keywords:** Muller’s Ratchet, Asexual evolution, *Halicephalobus mephisto*, Heterozygosity, Parthenogenesis, Extremophile adaptation, Genomics

## Abstract

Asexual reproduction often leads to loss of genetic diversity, but several mechanisms have evolved to maintain heterozygosity. The subterrestrial nematode, *Halicephalobus mephisto*, reproduces parthenogenetically, and here, we investigate how its genetic diversity $-$1.15% SNP heterozygosity—is retained from generation to generation. To test for loss of heterozygosity, we PCR-typed 56 individual animals at two different loci; no homozygotes were observed in the population. Furthermore, whole-genome analysis of parent and progeny demonstrated no transition from heterozygote to homozygote across over 620,000 SNPs. Surprisingly, these SNPs are not uniformly distributed throughout the genome, as we find multiple tracts of loss of heterozygosity (LOH) where no variation exists. Covering 4.3 million base pairs (Mb) genome-wide, these LOH tracts are most consistent with a recent meiotic recombination event or an error of chromosomal segregation. Supporting this, we observed chromosomal associations during reproduction that may reflect some degree of synapsis, potentially enabling recombination. However, full-genome analysis of parent–progeny pairs shows the current state of the genome remains stable, with no new LOH detectable, suggesting that history of *H. mephisto* is more dynamic than previously appreciated, and that heterozygosity has not always been retained with perfect fidelity. The potential functional and evolutionary consequences of this observation are discussed along with potential mechanisms contributing to this unusual genomic history.

**Supplementary Information:**

The online version contains supplementary material available at 10.1007/s00239-025-10259-3.

## Introduction

The maintenance of sexual reproduction in most eukaryotic organisms is a scientific mystery because asexual reproduction is more efficient. Sexual reproduction imposes the “cost of males” (Gibson et al. 2017): individuals who contribute little to the next generation, yet who consume resources in their production. The fact that sexual reproduction is widespread suggests it has a counterbalancing benefit. Many theories have been proposed, but the most common are that sexual reproduction promotes adaptive changes by novel allelic combinations, or that it promotes the elimination of deleterious mutations (Butlin 2002).

Asexual reproduction therefore is of significant scientific interest. Some postulate that long-lasting asexual lineages should not exist: a model known as Muller’s Ratchet (Muller 1964; Felsenstein 1974; Kondrashov 1994). This model describes an irreversible buildup of harmful mutations in asexual populations or those with minimal genetic recombination. Often described as a kind of irreversible ratchet mechanism, it fates small populations of asexual organisms with genomic meltdown and certain extinction as mean fitness decreases (Duarte et al. 1992; Gabriel et al. 1993; Lynch and Gabriel 1990; Matuszewski et al. 2017). The concept of Muller’s Ratchet has been extrapolated to metazoan aging, where the accrual of mutations in somatic cell lineages parallels the irreversible accumulation of mutations seen in asexual populations (Govindaraju et al. 2020).

Given the paradigmatic nature of Muller’s Ratchet, it is surprising to find many asexual eukaryotic organisms exist, and some are thought to be ancient. These “genomic scandals” (Schön and Martens 2003; Judson and Normark 1996) are important scientifically. Animal asexual species include the Amazon molly fish *Poecilia formosa* (Loewe and Lamatsch 2008; Warren et al. 2018), Anolis lizards (Fujita and Moritz 2009), bdelloid rotifers (Lujano 2014; Colinas et al. 2023), freshwater ostracod crustaceans (Chaplin et al. 1994; Butlin et al. 1998), Daphnia (water fleas) (Ebert 2022), the nematodes *Diploscapter pachys* (Fradin et al. 2017), *Diploscapter coronatus* (Hiraki et al. 2017), and *Mesorhabditis belari* (Blanc et al. 2023), the clonal raider ant *Ooceraea biroi* (Lacy et al. 2024) aphids (Davis et al. 2021), and the subterrestrial nematode *Halicephalobus mephisto* (Borgonie et al. 2011). Additional examples can be found among plants (Hörandl 2024).

If an organism becomes truly automictic, or lacking any form of meiosis and recombination, its reproduction is clonal (Neiman et al. 2014) and the two allelic haplotypes will diverge as they accumulate neutral or mildly deleterious mutations, becoming increasingly divergent over time (Butlin 2002). This phenomenon is known as the Meselson effect and leads to high heterozygosity (Butlin 2002). However, it is more common for asexual organisms to deploy a modified meiosis, or automixis, in which the production of diploid offspring occurs through fusion of meiotic products (Neiman et al. 2014). When this occurs, loss of heterozygosity (LOH) generally occurs if recombinant and non-recombinant chromosomes co-segregate (Neiman et al. 2014). LOH can be problematic for organisms, whether sexual or asexual, if it allows recessive deleterious alleles to negatively impact fitness (Archetti 2010; Bothwell et al. 2010; Heil 2023). At the extreme, gamete replication—the endoreplication of one meiotic product—leads to LOH genome-wide (Mirzaghaderi and Hörandl 2016) and is employed by whiptail lizards (Ho et al. 2024), and a wide range of asexual arthropods (Ma and Schwander 2017), among others.

In light of the risk of LOH, some organisms have modified apomictic reproduction specifically to retain genetic diversity. Two recent studies showed that the asexual nematode *Mesorhabditis belari* and the asexual clonal raider ant *Ooceraea biroi* both retain genomic recombination, but non-random segregation of recombined chromatids reunites complementary recombinant chromosomes, ensuring that essentially no LOH occurs (Blanc et al. 2023; Lacy et al. 2024). This is a non-selfish meiotic drive, and these discoveries point toward unexplored mechanisms.

Here, we analyze *H. mephisto*, a subterranean nematode discovered 1.3 km beneath the Earth’s surface in a South African goldmine (Borgonie et al. 2011). Native to an extreme habitat characterized by elevated temperatures (37 $^{\circ }$C), low oxygen levels, and the presence of methane, this organism displays evolutionary adaptations including enhanced stress responses (Weinstein et al. 2019) and thermally responsive mitochondria (Guerin et al. 2024). It reproduces through parthenogenesis, as single larvae develop into female individuals that give rise to entire populations; no sperm were detected in over 150 individuals, and no testes and no males were ever observed (Borgonie et al. 2011) (and personal communication, G. Borgonie). Because *H. mephisto* has been estimated to have separated from the lineage leading to *C. elegans* between 22 and 100 million years ago (Weinstein et al. 2019), this organism is a scientifically important model. Here, we show that *H. mephisto* faithfully maintains heterozygosity at over 620,000 SNPs genome-wide, but paradoxically has undergone recent LOH in large tracts of the genome. Our work documents an episodic history of heterozygosity accumulation and loss in *H. mephisto*.

## Results

**Fig. 1:**
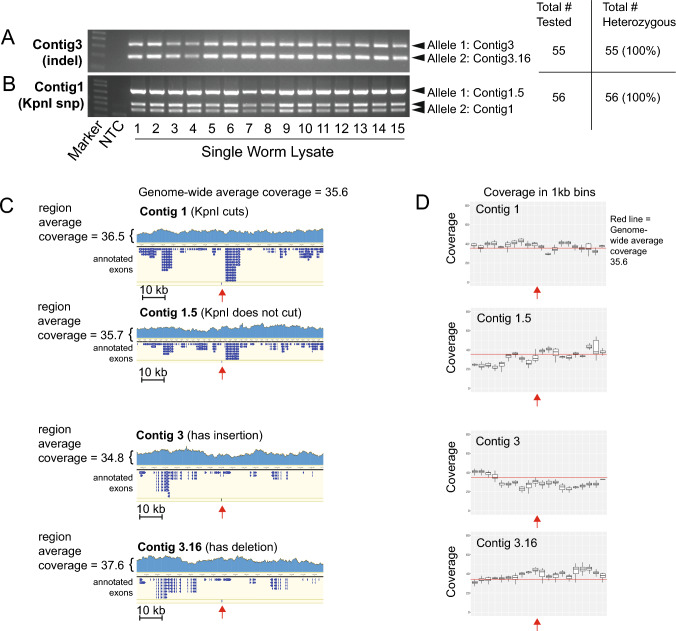
Single-worm lysis and PCR technique for genotyping of two loci in *Halicephalobus mephisto* using single-worm lysates. **A** PCR results showing heterozygosity for a naturally occurring indel on Contig3. **B** PCR results for a KpnI restriction-enzyme SNP on Contig27

Over time, heterozygosity in asexual organisms can be maintained, partly lost, or completely lost (Neiman et al. 2014). We therefore decided to check for maintenance of heterozygosity in individual *H. mephisto*. We optimized a single-worm lysis and PCR protocol specifically for *H. mephisto*, adapting a previously published protocol (see Methods). From our culture, we randomly isolated 56 single nematodes and PCR tested them at two loci: Contig1/Contig1.5 and Contig3/Contig3.16 allelic pairs. The Contig3 and Contig3.16 locus is a naturally occurring allelic indel yielding two band sizes easily distinguished by agarose gel electrophoresis; all animals tested were heterozygotes for this locus (Fig. 1A). Similarly, a KpnI restriction-enzyme single-nucleotide polymorphism (SNP) on Contig1 and Contig1.5 was always found in heterozygous condition (Fig. 1B). We validated the genomic loci by read coverage analysis, confirming each allele represents a single copy in the genome (Fig. 1C and D). Our data show that heterozygosity is maintained at least for these two loci.

To expand our findings genome-wide, we developed a population-based PCR-free method to analyze LOH in which single worms were isolated and cultured for 3–5 generations to generate sufficient genetic material for sequencing. Nanopore libraries were constructed using the PCR-free Rapid Barcoding direct genomic DNA sequencing kit, and the genomic data were mapped onto a single haplotype. Using bcftools, we called single-nucleotide polymorphism (SNP) variants relative to that haplotype. The alternative allele frequency is an estimate of heterozygosity at each location, with variation based on the actual read coverage at each site (which is stochastic) but should be close to 50% for heterozygous sites; LOH is detected either as 100% alternative allele frequency or as a lack of SNP calls (0% alternative allele frequency).

**Fig. 2:**
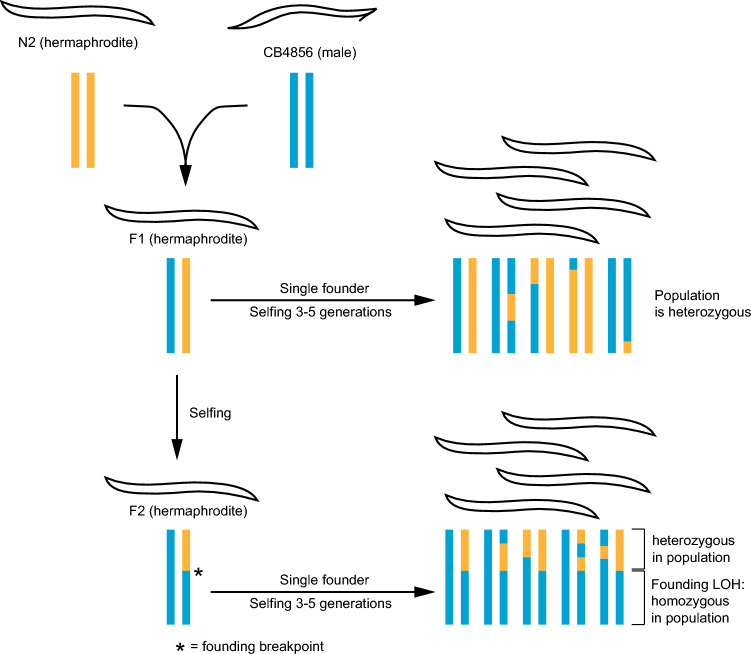
Schematic showing experimental design for detecting LOH events in population sequencing data. Single worms are isolated for F1 and F2 and allowed to grow 3–5 generations before population harvest and sequencing

To obtain sufficient DNA to perform PCR-free sequencing, we needed to grow nematodes into a population from a single founder animal. This raises concerns about potential LOH occurring during culture and obscuring actual heterozygosity in the population. While at a population level both heterozygosity and LOH in the founder should be detectable (Fig. 2), we wanted to empirically test this, so we designed a control experiment. Given that *C. elegans* are highly inbred and homozygous (unlike *H. mephisto*), we created a cross that would set up a high rate of heterozygosity genome-wide in an F1 animal. We crossed the standard N2 strain with the Hawaiian CB4856 strain to generate F1 cross-progeny, which was then singled onto its own plate. From this F1 animal, we subsequently isolated five separate F2 animals (direct progeny of the F1) onto their own plates. The isolated F1 and F2 animals were cultivated at 20 °C for 11–15 days (between 3.7 and 5 generations) prior to DNA extraction and analysis. After sequencing to a depth of 21–54× coverage, the data were mapped onto the N2 genome, identifying CB4856 as alternative SNP calls. Our method is sensitive: we identified 359,658 SNPs; a previous study reports 327,050 (Thompson et al. 2015).

**Fig. 3:**
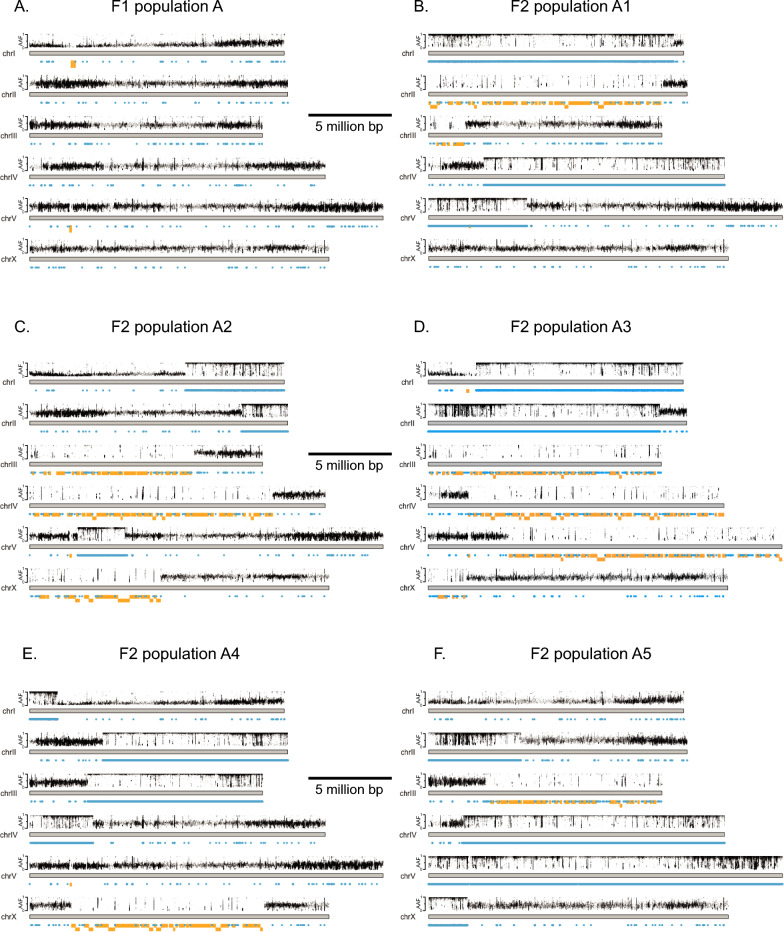
Plots of *C. elegans* heterozygosity plotted as alternative allele frequency (AAF) above each chromosome. Blue or gold color indicate homozygous CB4856 or N2, respectively, and are detected either as AAF of 1.0 or by a lack of SNPs in at least a 100 kb block (homozygosity of 0). Data are from populations derived from **A** F1 cross-progeny from N2 and CB4856 (Hawaiian) strains, (**B**–**F**) F2 individuals from that F1 (Color figure online)

We hypothesized that the population derived from the F1 individual should display 50% allelic heterozygosity across the entire genome, and reassuringly, this is what the data showed (Fig. 3A), consistent with theory (Fig. 2). In contrast, the F2-derived populations demonstrated varying recombination events, exemplified by LOH either to alternative allele frequencies of 1.0 at sites of homozygous Hawaiian alleles or a significant loss of SNP calls in regions that become homozygous N2; these regions are no longer called as SNPs by the bcftools pipeline (Fig. 3B–F). We note that recombination events are detectable on nearly every chromosome, and that the recombination pattern is distinct in each F2 population (Fig. 3B–F).

Having validated our method, we used it to measure recombination in *H. mephisto*. A single *H. mephisto* nematode was isolated from our established culture (P3), from which two direct descendants (P3.1 and P3.3) were subsequently isolated. To isolate sufficient DNA, the parental animal (P3) and its progeny (P3.1 and P3.3) were cultivated for one week at 37 $^{\circ }$C (three generations (Guerin et al. 2024)). We sequenced these three DNA samples to coverage depths of 21× for P3, 25× for P3.1, and 25× for P3.3, respectively. The reads were mapped onto a haploid (primary) genome assembly to enable detection of the other haplotype as alternative SNP calls. Our analysis revealed 629,512 SNPs in the parental organism (P3), while 675,904 and 680,284 SNPs were identified in P3.1 and P3.3, respectively. In our previous, work we reported 707,190 SNPs (Weinstein et al. 2019) using Illumina data, consistent with the Nanopore analysis presented here.

**Fig. 4:**
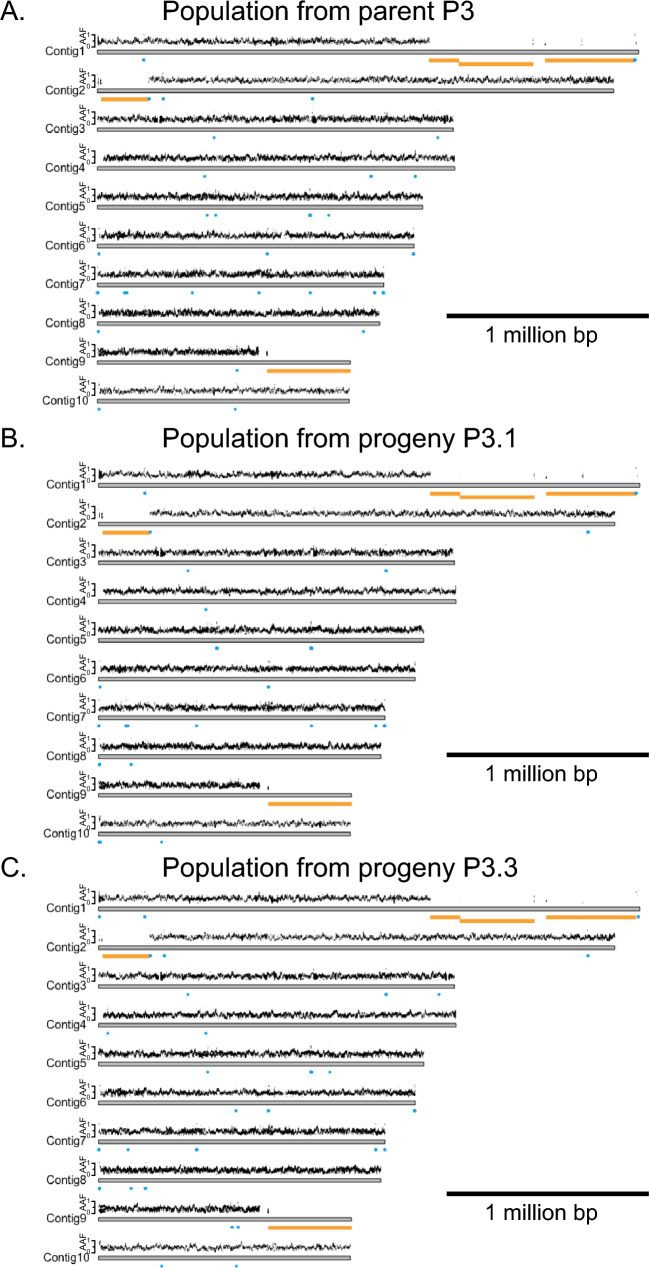
Plots of *H. mephisto* heterozygosity represented as alternative allele frequency (AAF) in populations derived from **A** parent P3, **B** direct progeny P3.1, and **C** direct progeny P3.3. Only the first 10 contigs are shown for readability. Two homozygous states are possible: homozygous alternative or homozygous reference. These are marked with blue or gold, respectively, in the track under the plot of alternative allele frequencies (Color figure online)

We performed the same heterozygosity analysis as for *C. elegans* but our analysis is more restricted because the genome of *H. mephisto* remains fragmented with a primary assembly N50 of 951 kb across 123 contigs and with the longest contig being 2,722,117 bp. Therefore, we plot Contigs 1–10 (the top 10 contigs by length) for this analysis (Fig. 4).

**Fig. 5:**
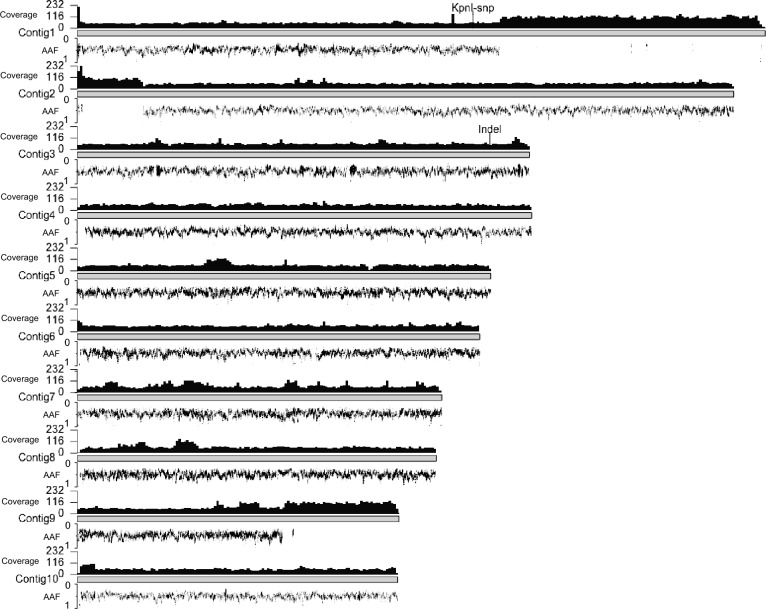
Combined Nanopore coverage from P3, P3.1, and P3.3 shown in track above contigs, and heterozygosity as alternative allele frequency (AAF), shown below contigs. Reads were mapped to diploid genome assembly to accurately visualize copy number. Notice that long regions lacking heterozygosity in Contigs 1, 2, and 9 also have twice the coverage of the remaining genome suggesting they are collapsed homozygous alleles. The sites of KpnI SNP and indel (from Fig. 1) are shown

From Fig. 4, it is apparent that heterozygosity remains consistent across all contigs in all samples, from P3 to P3.1 to P3.3. There is no shift from heterozygote to homozygote evident in either of these plots. However, another striking feature was revealed by this analysis: regions of apparent homozygosity present along Contigs 1, 2, and 9, which have no SNP calls at all and are reminiscent of the N2-homozygous regions identified in sexually recombining N2 (Fig. 3); these are present in both the parent and the progeny suggesting they pre-exist in our population. These regions might reflect an ancestral LOH event which is now locked into the parthenogenetically reproducing nematode genome. In that case, we predict (1) that they reflect two collapsed alleles, with double the coverage of a single allele (when mapped onto a diploid genome assembly); (2) that the candidate LOH regions lack an “alternative” contig in the assembly, given that they represent both alleles. Both these are true. The coverage of candidate LOH regions is twice the rest of the genome in diploid assembly mapping (Fig. 5). Furthermore, for the candidate LOH regions of Contigs 1, 2, and 9, there is no alternative contig in the genome assembly (Fig. S1). All LOH tracts (over 100kb) we identified in the haploid genome mapping account for 4.3 million base pairs, cumulatively, from a haploid genome size of 61.4 Mb (Weinstein et al. 2019), suggesting that 7% of the genome is homozygous.

To go beyond ten contigs to a whole-genome analysis, we developed a genome-wide LOH detection method. In this approach, we directly compare the alternative allele frequencies between a parent and progeny population; data are presented in a scatter-density plot. This should capture every LOH event at base pair resolution, with the caveat that it can only detect LOH events producing alternative alleles (reference homozygosity will not be called by bcftools as a variant). To avoid erroneous variant calls, we imposed a quality cutoff of 70 or above, which is stringent but not excessive. To avoid repetitive sequences, we also imposed a maximum coverage cutoff of 50× for *H. mephisto* sequences (which were sequenced to 21–25× coverage) and 100× for *C. elegans* (sequenced to 21–54× coverage). The only *H. mephisto* sequences not suitable for this method would be the 24% of the genome composed of repetitive sequences (Weinstein et al. 2019). Thus, our LOH detection method should survey the 76% non-repetitive genomic sequence of *H. mephisto*.

**Fig. 6:**
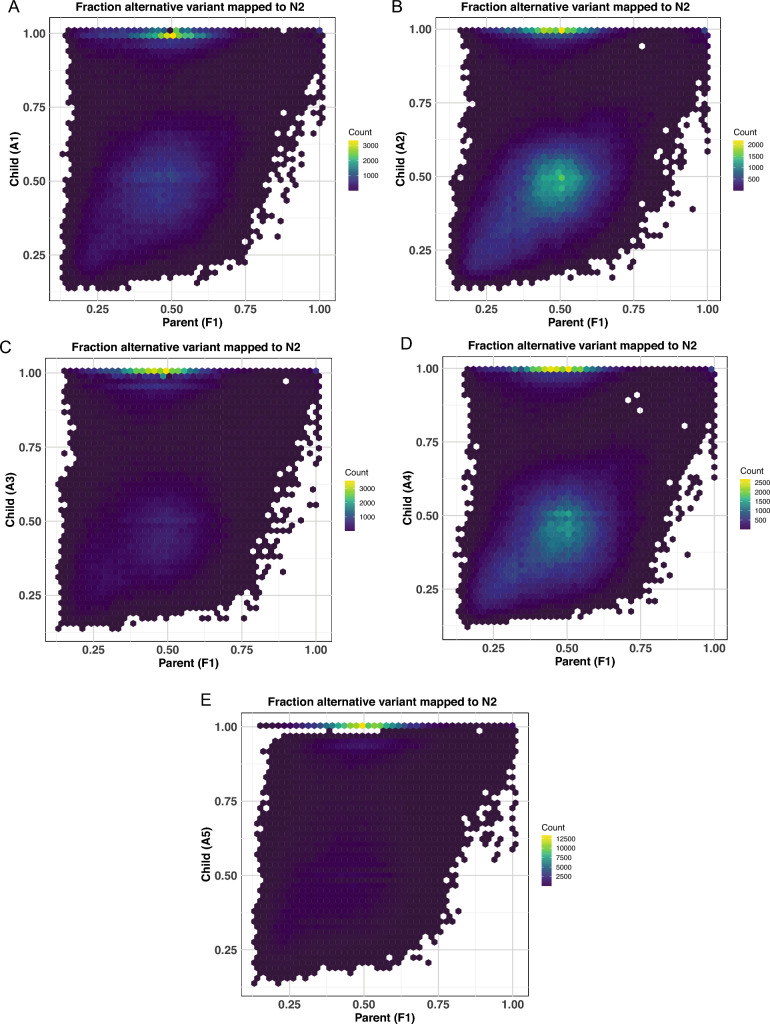
Alternative allele frequency (AAF) comparisons between parent and progeny in *C. elegans*. All plots show the alternative allele frequency (AAF) comparing the same locations (SNPs) from two datasets, from a population derived from the F1 animal on X-axis and the population derived from an individual F2 on the Y-axis. **A** F1 vs. F2 (A1). N = 255,337 shared SNPs shown. **B** F1 vs. F2 (A2). N = 270,356 shared SNPs shown. **C** F1 vs. F2 (A3). N = 166,313 shared SNPs shown. **D** F1 vs. F2 (A4). N = 320,956 shared SNPs shown. **E** F1 vs F2 (A5). N = 301,704 shared SNPs shown

**Fig. 7:**
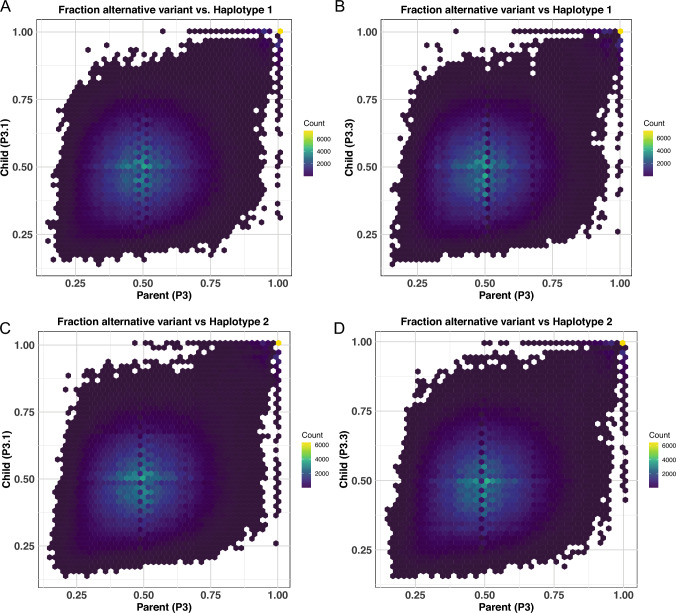
Alternative allele frequency (AAF) comparisons between parent and progeny in *H. mephisto*. All plots show the alternate alternative frequency (AAF) comparing the same locations (SNPs) from two datasets: the population derived from the parent (P3) animal on the X-axis and the population derived from a single progeny on the Y-axis. **A** Comparison of Child (P3.1) vs Parent (P3), both mapped onto Haplotype 1. N = 581,771 shared SNPs shown. **B** Child (P3.3) vs Parent (P3), both mapped onto Haplotype 1. N = 584,074 shared SNPs shown. **C** Child (P3.1) vs Parent (P3), both mapped onto Haplotype 2. N = 524,362 shared SNPs shown. **D** Child (P3.3) vs Parent (P3), both mapped onto Haplotype 2. N = 526,928 shared SNPs shown

To validate this method, we first performed the analysis with the *C. elegans* datasets, confirming that high levels of LOH are detected in the five F2 populations but not their F1 ancestor (Fig. 6). Specifically, we identify many SNPs with alternative allele frequencies of 0.5 in F1 and 1.0 in F2 (Fig. 6). However, a very different picture emerges in *H. mephisto* where allele frequencies are very close to 0.5 for both parent and progeny and any allele frequencies of 1.0 in progeny are also 1.0 in the parent (Fig. 7).

A significant advantage of this method is that it is minimally restrictive; it requires bcftools to identify the SNP in both datasets and then simply plots the alternative allele frequencies identified in both datasets. Therefore, any SNP that is called is captured in this analysis. It is truly genome-wide. However, one potential blind spot of the method is that if a region becomes homozygous “reference,” it would be lost as a non-call. (This concern is not debilitating; as we show with the *C. elegans* dataset, LOH to homozygous N2 clearly occurs and leads to reduced SNP calls in some regions (Fig. 3); nevertheless, the pipeline reveals significant LOH to homozygous CB4856 (Fig. 6)). However, we chose to eliminate this concern in *H. mephisto* by mapping reads onto Haplotype 2 (the alternative contigs) and calling variants. This approach will reveal any SNPs that might have become homozygous for Haplotype 1 (as now they would be homozygous alternative allele, at 1.0 on the plots). Nevertheless, this additional analysis also failed to identify LOH in the comparisons between P3 and P3.1 (Fig. 7C) or P3 and P3.3 (Fig. 7D).

**Fig. 8:**
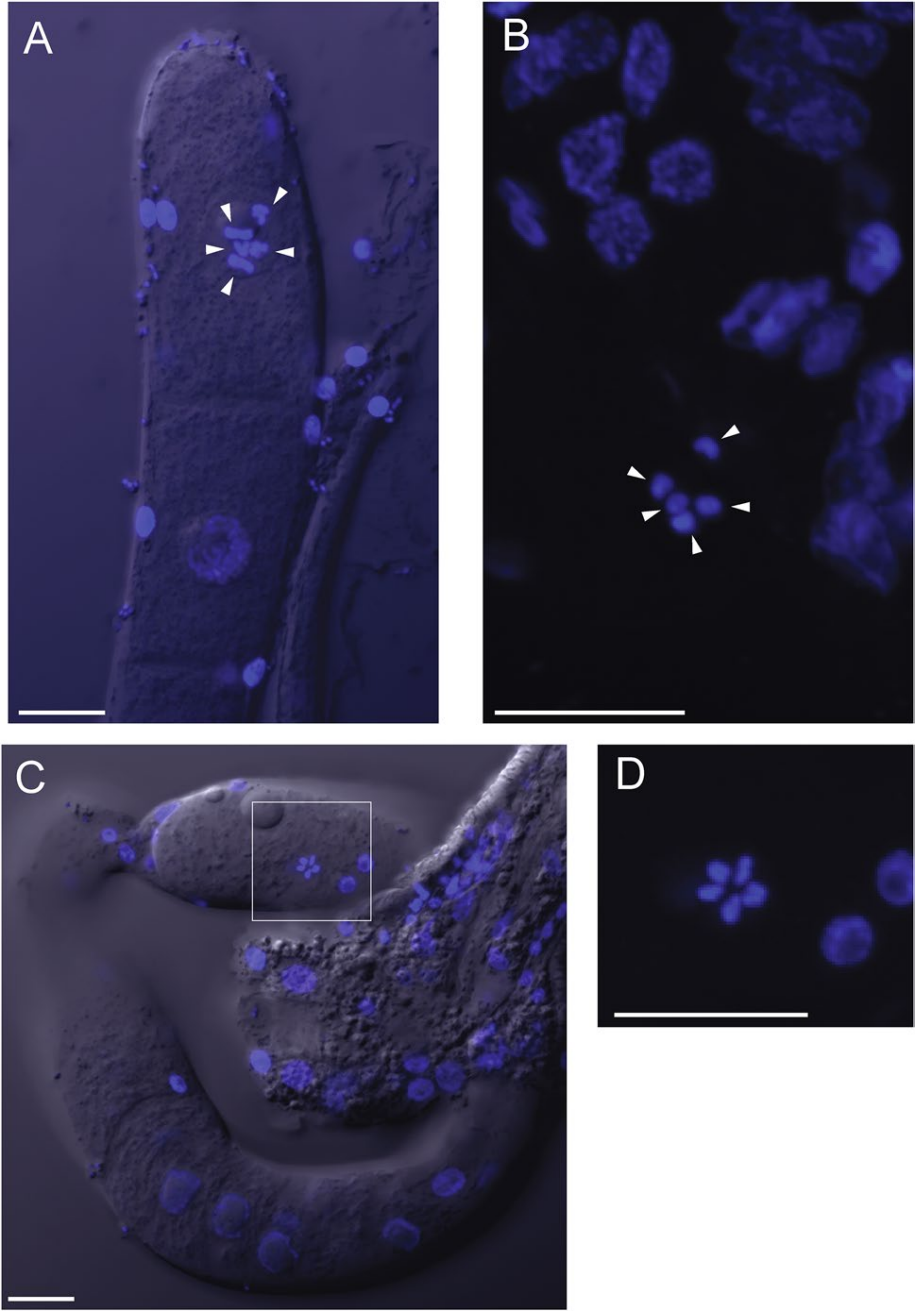
Visualization of chromosomal bivalents in *H. mephisto*. **A** and **B** Germline tissue showing five bivalents in a late meiotic oocyte (white arrowheads). **C** and **D** Early egg *in utero* revealing the same five bivalents in meiotic metaphase I

If sexual recombination occurred in the (relatively recent) past, does *H. mephisto* retain the ability to pair its chromosomes into bivalents? To investigate this, we analyzed dissected *H. mephisto* germline tissue, following a previously published method (Fradin et al. 2017). We found that the terminal oocytes display n = 5 highly condensed bivalents (Fig. 8A and B). Additionally, an early-stage egg observed *in utero* also exhibited five bivalents, arranged in what appeared to be meiotic metaphase I (Fig. 3C and D). These cytological observations indicate a chromosomal complement of 2n = 10 in *H. mephisto*. The observed meiotic chromosomal associations may reflect some degree of synapsis, suggesting that recombination events might be able to occur and potentially explaining the recombination and LOH event which has occurred in the recent history of this organism.

We asked whether the genes involved in meiotic recombination are present in *H. mephisto*. As an initial assessment, we performed a BLASTp analysis of 23 meiotic recombination genes (BLM, CHK1, DMC1, EXO1, HIM5, HIM8, HIM14, HIM17, HUS1, MLH1, MSH4, MSH5, NBN, PMS2, RAD51, RAD52, RAD54, RFA1, SLX1, SPO11, STX3, STX5, and SYCP1) within *H. mephisto* predicted proteins. In all cases, if *C. elegans* proteins were available, they were used as query; otherwise, *D. melanogaster* or *H. sapiens* were used as alternates (in that order of preference). To our surprise, 16 of the 23 were detectable by BLASTp to an evalue of 1e-5. While HUS1 was not originally identified by BLASTp, pfam domain PF04005 (denoting a HUS1-like protein) was identified by HMMER (Eddy 2011) at an e-value of 2e-11 (Table 1). We used HMMER to search for NBN, SYCP1, and RAD52 and no matches were identified. Hidden Markov model files are not available for HIM5, HIM8, or HIM17 so we were not able to search for them using this method. Therefore, we successfully identified a total of 17 of 23 meiotic recombination proteins (74%) (Table 1). We failed to identify RAD52, NBN SYCP1, HIM5, HIM8, and HIM17. Interestingly HIM8 was also undetected in asexually reproducing *D. pachys* (Fradin et al. 2017). Given the evolutionary divergence between *D. pachys*, a Clade V nematode, and *H. mephisto*, a Clade IV animal, we assume independent losses of HIM8 in these lineages.

At least two concerns must be noted in this analysis. First, BLASTp is not a highly sensitive method for highly divergent protein identifications, and *C. elegans* and *H. mephisto* have diverged by at least 22 million years as a best estimate (Weinstein et al. 2019). This could contribute to the apparent lack of some meiotic genes. Consistent with this, while HUS1 was not detected by BLASTp, a search with the more sensitive hidden Markov model scanning software HMMER (Eddy 2011) found a good match, suggesting that evolutionary divergence may be responsible for the lack of identification by BLASTp. However, as noted, HMMER also did not identify the other six proteins, suggesting they might be even more divergent in sequence, be truly missing, or be missing for other reasons.

The second concern is that these protein genes belong to extensive families of functionally related proteins (Spo11, for instance, constitutes a member of the topoisomerase VI family), thereby introducing the possibility that our analysis may have identified paralogous rather than orthologous sequences. A more thorough bioinformatic analysis will be required to definitively conclude what meiotic recombination proteins are retained in *H. mephisto*, but it does suggest that at least some of the recombination machinery is present.

## Discussion

LOH can occur by sexual recombination, but our data cannot rule out other LOH mechanisms, such as mitotic recombination (Chumki et al. 2016) or gene conversion (Chen et al. 2007). However, these mechanisms tend to produce shorter tracts of LOH, with mitotic recombination up to 150 kb (Chumki et al. 2016) and gene conversion considerably less (200–1000 bp) (Chen et al. 2007). Yet, here we show in *H. mephisto* a tract of LOH over 1 million base pairs (Mb) (Fig. 4), summing to 4.3 Mb genome-wide. The most parsimonious explanation of our data is a recent, but rare meiotic recombination event in a recent ancestor that left a permanent imprint on all its descendants.

Our results lead to two, paradoxical conclusions: (1) ongoing LOH is not occurring in the genome of *H. mephisto*, but (2) ancestrally, LOH did occur. Because the homozygous tracts are essentially identical (i.e., few or no new mutations have accumulated to differentiate the two alleles), we suspect that this ancestral recombination must have occurred relatively recently, in an ancestor whose genome already contained high SNP heterozygosity (over 620,000 SNPs).

The origin of over 620,000 SNPs between haplotypes in the *H. mephisto* genome presents another puzzle. We suggest two possibilities. First, high standing variation in a large sexual population prior to the origin of parthenogenesis could account for the 1.15% allelic diversity. Second, the high heterozygosity may reflect a gradual buildup over time after the origin of parthenogenesis (the Meselson effect (Ceplitis 2003)). Neither scenario can be dismissed based on our data. While 1.15% SNP density is high for a sexually reproducing species, some marine species have been reported with this level of diversity between haplotypes, for example, the sea squirt *Ciona intestinalis* (Kim et al. 2007) and the Pacific oyster *Crassostrea gigas* (Hu et al. 2022), among others. On the other hand, a buildup of 620,000 SNPs from a completely homozygous starting point can be achieved in a relatively short time given the rapid reproductive rate of nematodes. If one assumes a mutation rate of $3.4 \times 10^{-9}$ mutations per site per generation (Liu et al. 2017), then for *H. mephisto*, the per-genome, per-generation mutation rate is 0.2 (or one mutation in five generations), and 3.1 million generations are sufficient to accumulate 620,000 SNPs. Under optimal growth conditions, one *H. mephisto* generation occurs in 2 days (Guerin et al. 2024). Thus, the Meselson effect only requires between 17,000 years (at maximal 182.5 generations per year) or at the high end 310,000 years (at 10 generations per year, a conservative estimate). Since the divergence of *H. mephisto* and *C. elegans* is estimated to have occurred between 22 and 100 million years ago (Weinstein et al. 2019), ample time has elapsed for *H. mephisto* to accumulate 620,000 SNPs.

Asexual reproduction can occur in myriad ways, with different impacts on heterozygosity, from its preservation to its complete loss (Engelstädter 2017). For example, true clonal reproduction (apomixis) involves loss of meiosis and conserves heterozygosity perfectly (Mirzaghaderi and Hörandl 2016). We suspect *H. mephisto* does not reproduce by apomixis because of the LOH tracts in its genome and the fact that chromosomal pairing still occurs. Automixis, in contrast to apomixis, involves modified meiosis, wherein fusion of different meiotic products restores diploidy (Mirzaghaderi and Hörandl 2016). However, most automictic mechanisms involve substantial loss of heterozygosity when recombination occurs (Mirzaghaderi and Hörandl 2016; Engelstädter 2017). Fusion of meiosis I products, or central fusion, maintains heterozygosity proximal to the centromere; fusion of meiosis II products (terminal fusion) maintains heterozygosity distal to the centromere and produces higher overall homozygosity than central fusion automixis, but both produce substantial reductions in heterozygosity (Mirzaghaderi and Hörandl 2016; Engelstädter 2017). At the extreme, gamete replication—the duplication of the entire genome of a haploid meiotic product, restoring diploidy—produces 100% homozygous offspring (Mirzaghaderi and Hörandl 2016). This mode of reproduction has been documented for whiptail lizards (Ho et al. 2024) and many cases of endosymbiont-driven parthenogenesis in arthropods (Ma and Schwander 2017). Either gamete duplication or terminal fusion automixis are hypothesized in the stick insects (Jaron et al. 2022) and American cockroach (Tanaka et al. 2018), given that they display essentially complete LOH compared to sexual relatives.

Recent work has uncovered novel mechanisms retaining heterozygosity even when recombination occurs in automixis. For instance, the clonal raider ant *Ooceraea biroi* and the nematode *Mesorhabditis belari* both show an unusual form of automictic reproduction in which recombinant sister chromatids are co-segregated into the same offspring preferentially. This non-Mendelian chromatid assortment ensures that both parental alleles at each locus are transmitted together, preserving genome-wide heterozygosity despite frequent crossover events (Blanc et al. 2023; Lacy et al. 2024).

Retaining heterozygosity is thought to be important for asexual species to prevent inbreeding depression from deleterious recessive alleles (Archetti 2023). However, the opposite of heterozygosity maintenance—LOH—can also have important biological and evolutionary functions. In the clonal raider ant *Ooceraea biroi* and in brine shrimp *Artemia parthenogenetica*, LOH has been linked with the production of rare males when sex-determination loci lose their heterozygosity (Kronauer et al. 2012; Boyer et al. 2023; Lacy et al. 2025). Even more striking is the recently documented formation of a new queen-like mutant morph of clonal raider ant—a potentially new species—that is also linked with LOH (Trible et al. 2023). In light of these findings, the potential functional relevance of the LOH tracts in *H. mephisto* requires more research.

The presence of heterozygosity does not imply absence of recombination. From our data, we cannot rule out the possibility that *H. mephisto* uses a method akin to the raider ant *O. biroi* and nematode *M. belari*, in which recombination occurs regularly but heterozygosity is retained through preferential co-transmission of the recombinant chromosomes, thereby avoiding the loss of heterozygosity (Lacy et al. 2024; Blanc et al. 2023). If this is the case, then the large LOH tracts we note here (Fig. 4) reflect not an instance of recombination, but a failure to properly segregate the recombinant chromosomes together. This would also explain the apparent bivalent formation in reproduction (Fig. 8) and the presence of many meiotic genes in the genome (Table 1).

*Halicephalobus mephisto’s* genomic heterozygosity presents an apparent contradiction: it appears to be stable from generation to generation in our tests (Figs. 1, 4, and 7), yet it has been lost in multiple places (Fig. 4). The former usually is associated with asexual reproduction without recombination; the latter, with asexual reproduction in the presence of recombination. Given that heterozygosity can be retained along with recombination as long as meiosis is adjusted so that recombinant chromosomes co-segregate (Lacy et al. 2024; Blanc et al. 2023), perhaps *H. mephisto*’s LOH tracts reflect an error in segregation, not recombination. Whatever the cause, this event (the occurrence of sex, or errors in segregation, or chromosomal recombination) appears to have been transitory in the evolutionary history of *H. mephisto*. Open questions for future research include whether heterozygosity is stable in this species, whether transitions from heterozygosity-building into heterozygosity-reducing modes are triggered by environmental or developmental cues, and whether the effects of heterozygosity gain or loss have played functional evolutionary roles in the history of this intriguing organism.

**Table 1:** BLAST and HMMER results for candidate meiotic recombination genes in *H. mephisto*

Gene	Query accession	Search method	Query species	*H. mephisto * match	E-value
BLM	O18017	BLASTp	*C. elegans*	MSTRG.16394.1.p1	0.0
CHK1	Q9N3Z3	BLASTp	*C. elegans*	MSTRG.20910.1.p1	5e-108
DMC1	Q0E9B5	BLASTp	*D. melanogaster*	MSTRG.8861.9.p1	0.0
EXO1	O62245	BLASTp	*C. elegans*	HMEPH_g23347.t1.p1	3e-15
HIM5	D1086.4a	BLASTp	*C. elegans*	(Not found)	–
HIM8	T07G12.12a	BLASTp	*C. elegans*	(Not found)	–
HIM14	Q23405	BLASTp	*C. elegans*	MSTRG.5169.1.p1	3e-166
HIM17	T09E8.2	BLASTp	*C. elegans*	(Not found)	–
HUS1	G5EFI9	BLASTp	*C. elegans*	(Not found)	–
HUS1	Pfam PF04005	HMMER	Multi-species alignment	HMEPH_g2100.t1.p1	2e-11
MLH1	A0A087WX20	BLASTp	*H. sapiens*	HMEPH_g8240.t1.p1	3e-35
MSH4	O15457	BLASTp	*H. sapiens*	MSTRG.5391.1.p2	1e-147
MSH5	Q19272	BLASTp	*C. elegans*	HMEPH_g21649.t1.p1	2e-164
NBN	O60934	BLASTp	*H. sapiens*	(Not found)	–
NBN	Pfam PF08599 & PF16508	HMMER	Multi-species alignment	(Not found)	–
PMS2	G5EFG5	BLASTp	*C. elegans*	MSTRG.6743.1.p1	6e-68
RAD51	Q95Q25	BLASTp	*C. elegans*	HMEPH_g4285.t1.p1	2e-131
RAD52	Q5DR82	BLASTp	*H. sapiens*	(Not found)	–
RAD52	Pfam PF04098	HMMER	Multi-species alignment	(Not found)	–
RAD54	Q18241	BLASTp	*H. sapiens*	MSTRG.4488.6.p1	3e-145
RFA1	Q19537	BLASTp	*C. elegans*	HMEPH_g5328.t1.p1	6e-102
SLX1	P91351	BLASTp	*C. elegans*	MSTRG.9501.2.p2	2e-97
SPO11	Q22236	BLASTp	*C. elegans*	MSTRG.127.2.p2	6e-59
STX3	Q20024	BLASTp	*C. elegans*	HMEPH_g10434.t1.p1	5e-39
STX5	Q20797	BLASTp	*C. elegans*	MSTRG.4609.3.p1	2e-86
SYCP1	Q15431	BLASTp	*H. sapiens*	(Not found)	–
SYCP1	Pfam PF05483	HMMER	Multi-species alignment	(Not found)	–

## Methods

*Halicephalobus mephisto* culture. *H. mephisto* were cultured using standard *C. elegans* NGM growth media supplemented with OP50 *E. coli* Stiernagle (2006) but cultures were maintained at 37 °C in a microbial incubator. A founder parent animal was singled (by picking) onto its own plate (P3), and from its immediate progeny, P3.1 and P3.3 were singled (by picking) onto their own plates. These worms gave rise to populations which were harvested once they had starved their culture plates after 7 days of growth at 37 °C (3.5 generations since the lifecycle is 48 h (Guerin et al. 2024)). These populations were harvested by washing in M9 buffer Stiernagle (2006) twice, pelleting the worms at 400 g for 2 min followed by supernatant removal and addition of fresh M9 to wash. For freezing, the worms were pelleted and supernatant removed leaving 150 µL of M9 buffer, and 150 µL of DNA/RNA Shield Buffer (a component of the Zymo Quick DNA/RNA kit, Catalog number D7003) was added, followed by gentle mix and quick freezing at −80, where they were stored until DNA extraction. *C. elegans* culture. Both N2 and CB4856 were cultured using standard *C. elegans* NGM growth media supplemented with OP50 *E. coli*. Males were obtained by starvation of the CB4856 strain and crossed with N2 hermaphrodites. F1 cross-progeny were isolated and confirmed by single-worm qPCR following (Shelton 2006), and using the snp W10C8 primers from Table 1: Primer 1: 5’-ACTACATCCTCTCGTCATTTCTCAGTA-3’, Primer 2: 5’-GTCGTCTCTATTCAAATCTCCTCCCA-3’. This analysis confirmed that F1 individual “A” was a cross-progeny (both homozygous N2 and CB4856 were used as controls). From F1 A, we singled out five F2 for analysis: A1–A5. These worms were cultured at 20 °C for 15 days for F1 and 11 days for A1-A5. Because the *C. elegans* lifecycle is 3 days at 20 °C, the F1 population has undergone 5 total generations, while F2 populations have undergone 3.7 generations. The populations were harvested, pelleted, and frozen as described for *H. mephisto* until DNA extraction.

**DNA Extraction:** For DNA extraction, the Zymo Quick DNA/RNA extraction kit (Catalog number D7003) protocol previously described by Weinstein et al. (2019) was followed. The frozen pellet of 300 µL was thawed and added directly to a Zymo ZR BashingBead tube (Zymo catalog number S6012). The tube was taped directly onto the head of a vortexer and run at top speed for 3 min, followed by a 1 min break, and 3 more min vortex. The samples were digested in proteinase K buffer as described in the manufacturer’s instructions, with two modifications: the digestion was performed at 37 °C, and 20 µL of RNAse A (10 mg/mL) was added to remove genomic RNA. Zymo Quick DNA/RNA kit has a dual-column flow for isolating both nucleic acids; for this experiment, however, the RNA extraction was omitted and only DNA was collected. The DNA was eluted from the kit’s spin-column into 50 µL of nuclease-free water.

Once the DNA was extracted, it was again RNAse A treated. For this we added 10 µL of RNAse A stock (10 mg/mL) to the DNA (making 60 µL total) and incubated at 37 °C for 2 h. To clean up the DNA, we used the Zymo RNA Clean and Concentrator-5 (catalog number R1015), and the manufacturer’s instructions were followed for isolating large nucleic acid fragments. Briefly, the RNA binding buffer and 100% ethanol were pre-mixed prior to the addition of sample and loading onto the column. The samples were processed following manufacturer’s instruction including two washes with RNA wash buffer (ethanol) and a dry spin of 2 min prior to elution to remove residual wash buffer. Final DNA was eluted in 50 µL of nuclease-free water.

**Genome Sequencing and Bioinformatics for SNP Analysis:** The genomic DNA, prepared as described above, was converted into sequencing libraries using the Rapid Barcoding Kit (SQK-RBK114.24), and reads were generated using an Oxford Nanopore GridION. The raw data were mapped onto the haploid genome assemblies using minimap2 (version 2.28-r1209), and the variants were called using Bcftools (version 1.20) with the output of the VCF file. The VCF files were processed using custom Python scripts as a pipeline: parseVCF-freq2.py (to extract reference and variant frequency data from the VCF files) and then filter-text-files.py (to filter for only SNPs) and then compare-text-files.py (to compare parent to child populations by matching the SNPs). Plots were made in R (4.3.2) run in Rstudio (2023.09.1+494). Karyotype plots were created using the karyoploteR package (Gel and Serra 2017).

All computational methods detailed at the Bracht lab Githhub page https://github.com/brachtlab/mephisto-heterozygosity-analysis.

**Bioinformatics for Coverage Analysis:** The raw reads (combined P3, P3.1, and P3.3) were mapped onto the diploid genome assembly (containing alternative contigs) using minimap2 with the option—secondary = no to ensure only primary alignments were analyzed. Data were visualized from a sorted, indexed bam file using the kpPlotBAMDensity() function within the karyoploteR R package (Gel and Serra 2017). Plots were made in R (4.3.2) run in RStudio (2023.09.1+494). Computational details at the Bracht lab Githhub page https://github.com/brachtlab/mephisto-heterozygosity-analysis.

**Single-Worm Lysis and PCR:** Worms were cultured as described above, and the single worms were isolated and lysted in a custom lysis buffer. To make 500 µL lysis buffer, we combined 450 µL water, 50 µL 10× CutSmart buffer, and 25 µL 20 mg/mL Proteinase K. Single worms were picked into 15 µL of this lysis buffer added to the cap of 0.2-mL strip tubes for PCR. After singling one worm per cap, the lids were carefully closed and centrifuged to move the worms and lysis buffer to the bottom of the tube. The tubes were frozen at − 80 for at least 5 min (sometimes stored for months), and lysis was completed by running a worm lysis program in the PCR machine: 65 $^{\circ }$C for 60 min followed by 95 $^{\circ }$C for 10 min. This raw lysate was used in PCR at 3 µL per reaction. PCR programs were as follows: for Contig27SNP: 40 cycles of 94 $^{\circ }$C denature (15 s), 60 $^{\circ }$C annealing (30 s), and 72 $^{\circ }$C extension (50 s); products were digested with 1 µL KpnI for 30 min at 37 $^{\circ }$C prior to running the gel. For Contig3, PCR conditions were as follows: 40 cycles of 94 $^{\circ }$C denature (15 s), 52 $^{\circ }$C annealing (30 s), and 72 $^{\circ }$C extension (30 s). Because Contig3 amplifies an indel, no further processing is required before running a gel. All PCR products of were visualized on a 2% agarose TAE gel.

**BLASTp** *C. elegans* protein sequences were extracted from Uniprot (www.uniprot.org) and used in BLASTp analysis as a query against the *H. mephisto* predicated proteome generated by translating coding sequences from the genome annotation, with -evalue set to 1e-5.

**HMMER** Protein domains (e.g., PF04005) were downloaded from InterPro as.hmm files and used as queries for the ’hmmsearch’ command in HMMER version 3.4 (Eddy 2011).

**Microscopy of Chromosomes:** Gonads were isolated and examined as described Fradin et al. (2017). In brief, cover slips were pre-treated with 1 mg/mL polylysine (two applications of 10 µL, dried completely). Worms were washed in M9 twice and dissected on cover slips in a 30 µL with two 21 G 1 1/2 hypodermic needles to liberate gonads. After fixation in 2% formaldehyde for 5 min, they were incubated in 100% methanol for 20 min and washed in PBS prior to mounting with Glycerol Mounting Medium with DAPI (Electron Microscopy Services, Cat # 17989-60). Imaging was performed on an Olympus FV1200 scanning confocal microscope.

## Supplementary Information

Below is the link to the electronic supplementary material.

Supplementary file1 (PDF 1190 KB)
Fig. S1: LastZ alignment of primary and alternative contigs, for (A) Contig1, (B) Contig2, and (C) Contig9. For all plots, the primary contig is shown on X and the alternative contigs on Y, with their alignment as a dot plot. SNPs are shown in the annotations underneath each primary contig, with one color per base pair: purple (A-SNPs), brown (G-SNPs), blue (C-SNPs), and gold (T-SNPs)

## Data Availability

Raw genomic data available at sequence reads archive (SRA) bioproject accession PRJNA1167777. https://www.ncbi.nlm.nih.gov/bioproject?LinkName=sra_bioproject&from_uid=38884687.

## Code Availability

All code, documentation, and related genomic data available in Github page of Bracht lab, “mephisto-heterozygosity-analysis”. https://github.com/brachtlab/mephisto-heterozygosity-analysis.
