# Supplementary figures and images for: Gain and Loss of Heterozygosity in the Genome of the Asexual Nematode Halicephalobus mephisto
Source: J Mol Evol. 2025 Aug 6;93(4):511–26. doi: 10.1007/s00239-025-10259-3 (PMC12354626; doi:10.1007/s00239-025-10259-3)

A. Contig1

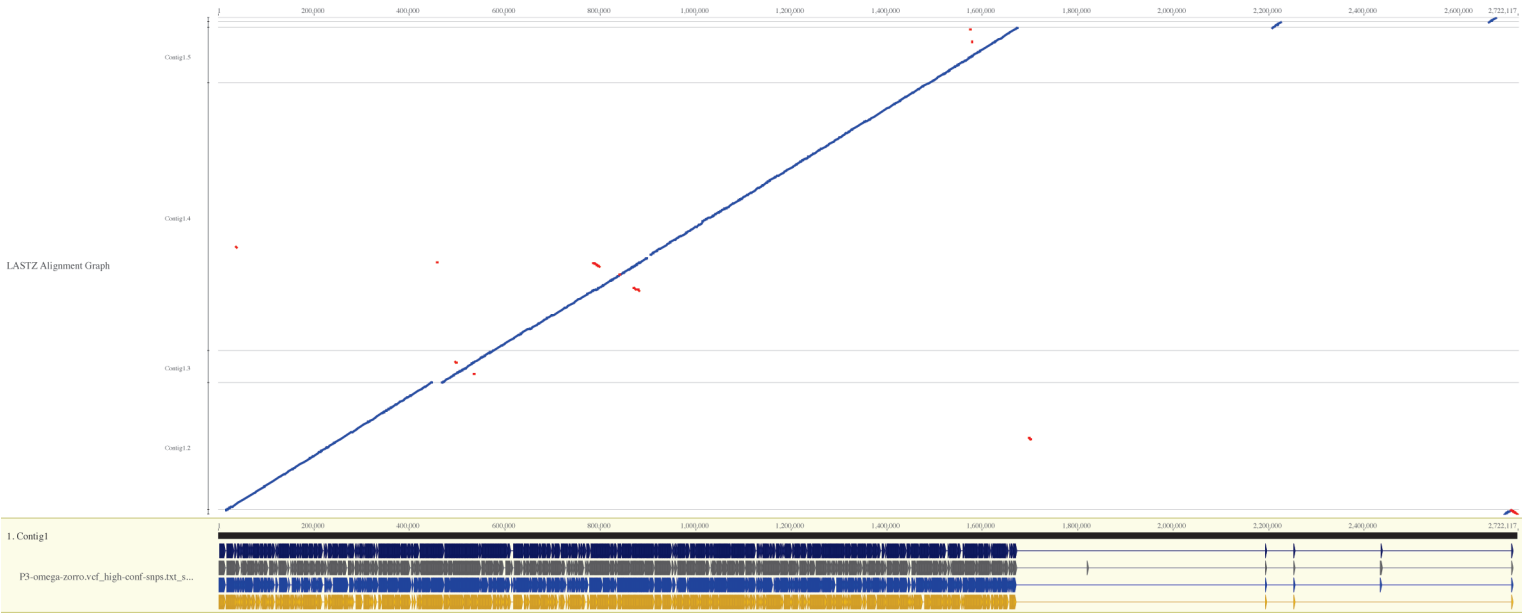

B. Contig2

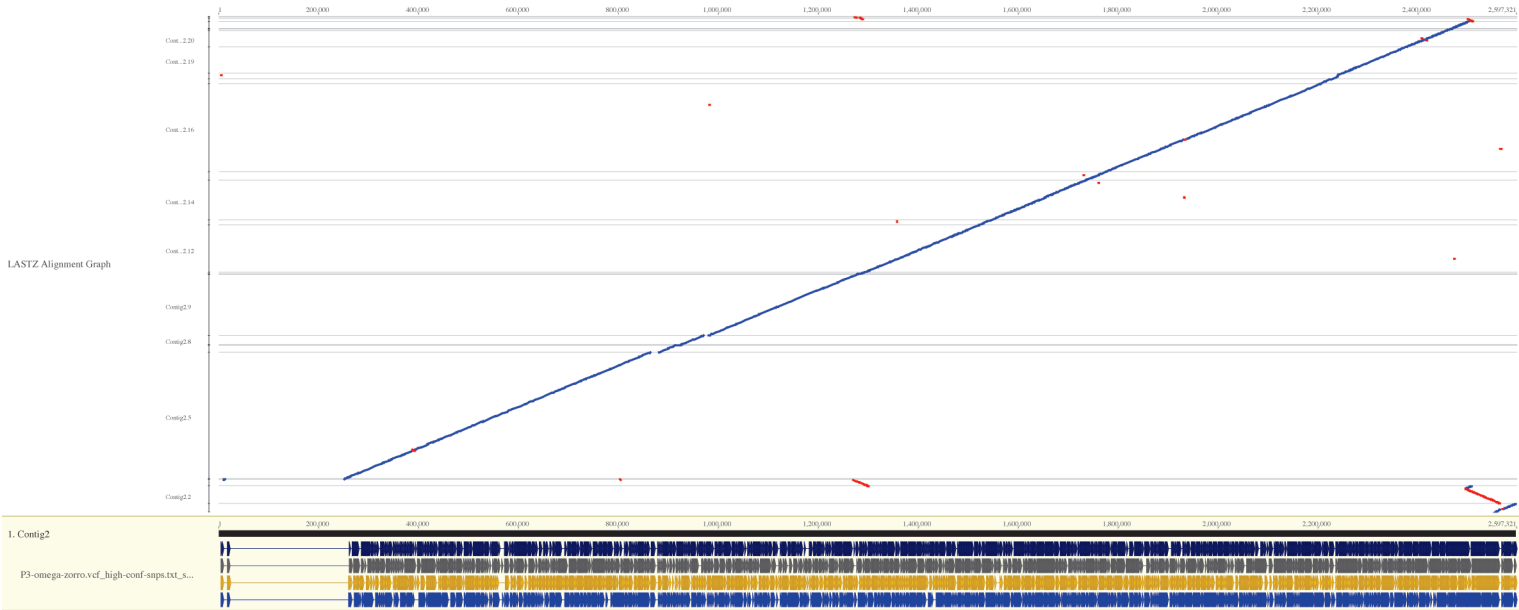

C. Contig9

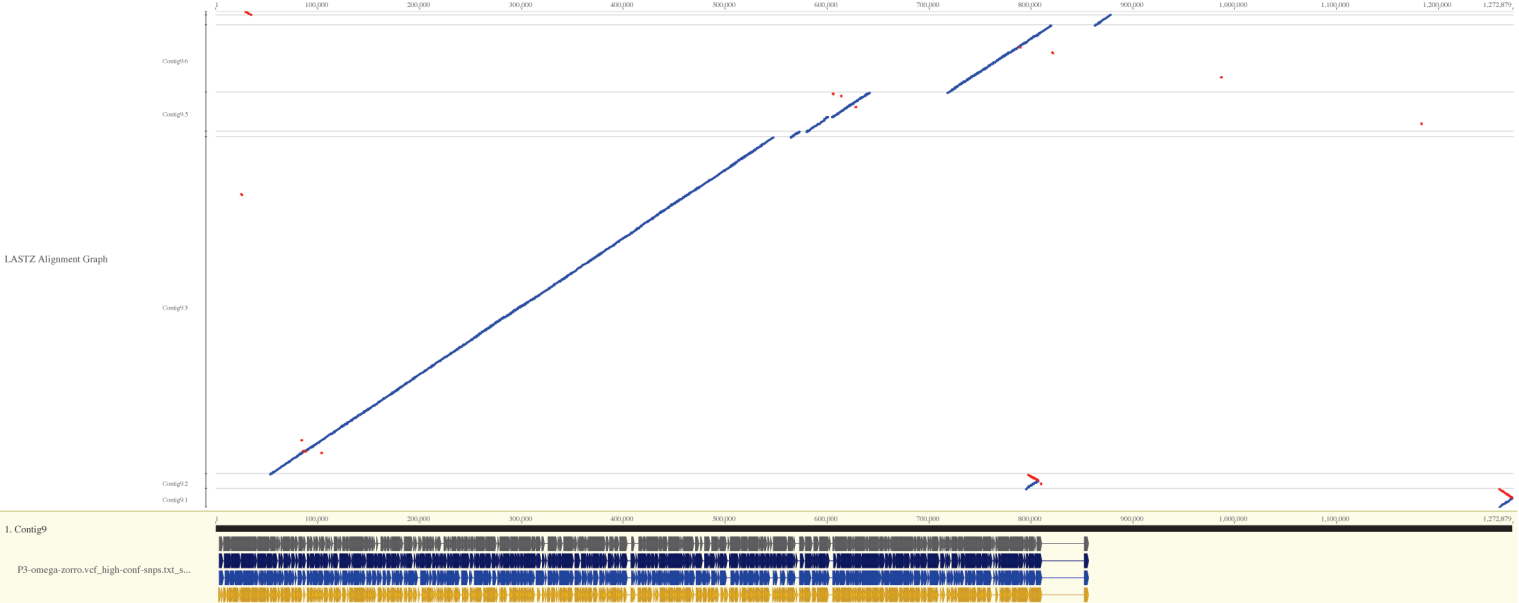

Supplement: Supplementary file 1 — Supplementary file1 (PDF 1190 KB) Fig. S1: LastZ alignment of primary and alternative contigs, for (A) Contig1, (B) Contig2, and (C) Contig9. For all plots, the primary contig is shown on X and the alternative contigs on Y, with their alignment as a dot plot. SNPs are shown in the annotations underneath each primary contig, with one color per base pair: purple (A-SNPs), brown (G-SNPs), blue (C-SNPs), and gold (T-SNPs) [file 239_2025_10259_MOESM1_ESM.pdf]
